# Loss of receptor activity-modifying protein 2 in mice causes placental dysfunction and alters PTH1R regulation

**DOI:** 10.1371/journal.pone.0181597

**Published:** 2017-07-20

**Authors:** Mahita Kadmiel, Brooke C. Matson, Scott T. Espenschied, Patricia M. Lenhart, Kathleen M. Caron

**Affiliations:** 1 Department of Cell Biology and Physiology, University of North Carolina at Chapel Hill, Chapel Hill, NC, United States of America; 2 Department of Genetics, University of North Carolina at Chapel Hill, Chapel Hill, NC, United States of America; Xavier Bichat Medical School, INSERM-CNRS - Université Paris Diderot, FRANCE

## Abstract

Receptor activity-modifying protein 2 (*Ramp2*) is a single-pass transmembrane protein that heterodimerizes with several family B G-protein coupled receptors to alter their function. *Ramp2* has been primarily characterized in association with calcitonin receptor-like receptor (*Calcrl*, CLR), forming the canonical receptor complex for the endocrine peptide adrenomedullin (*Adm*, AM). However, we previously demonstrated that *Ramp2*^*+/-*^ female mice display a constellation of endocrine-related phenotypes that are distinct from those of *Adm*^*+/-*^ and *Calcrl*^*+/-*^ mice, implying that RAMP2 has physiological functions beyond its canonical complex. Here, we localize *Ramp2* expression in the mouse placenta, finding that *Ramp2* is robustly expressed in the fetal labyrinth layer, and then characterize the effects of loss of *Ramp2* on placental development. Consistent with the expression pattern of *Ramp2* in the placenta, *Ramp2*^*-/-*^ placentas have a thinner labyrinth layer with significantly fewer trophoblast cells secondary to a reduction in trophoblast proliferation. We also find that absence of *Ramp2* leads to failed spiral artery remodeling unaccompanied by changes in the uterine natural killer cell population. Furthermore, we assess changes in gene expression of other RAMP2-associated G-protein coupled receptors (GPCRs), concluding that *Ramp2* loss decreases parathyroid hormone 1 receptor (*Pthr1*) expression and causes a blunted response to systemic parathyroid hormone (PTH) administration in mice. Ultimately, these studies provide *in vivo* evidence of a role for RAMP2 in placental development distinct from the RAMP2-CLR/AM signaling paradigm and identify additional pathways underlying the endocrine and fertility defects of the previously characterized *Ramp2* heterozygous adult females.

## Introduction

Receptor activity-modifying proteins (RAMPs) associate with G-protein coupled receptors (GPCRs) and enable the translocation of select GPCRs from the endoplasmic reticulum to the plasma membrane, ultimately affecting GPCR ligand bias and affinity, selectivity of downstream signaling pathways, and GPCR desensitization [1]. The pharmacologic tractability of GPCRs and, by extension, RAMPs emphasizes the importance of understanding these heterodimeric complexes [2, 3]. Since the majority of studies on RAMP-GPCR associations have focused on *in vitro* pharmacology, there is a need to assess the physiological and pathophysiological consequences of these interactions, particularly using genetically engineered mouse models [4].

RAMP2, the second of three mammalian RAMPs, has been well-characterized primarily for its association with calcitonin receptor-like receptor (*Calcrl*, CLR) as the canonical receptor for the endocrine peptide adrenomedullin (*Adm*, AM) [5–9]. Importantly, RAMP2 also binds other family B GPCRs: calcitonin receptor (*Ctr*), corticotrophin releasing hormone receptor 1 (*Crhr1*), glucagon receptor (*Gcgr*), parathyroid hormone receptor 1 (*Pth1r*), and vascoactive intestinal polypeptide receptor types 1 and 2 (*Vpac1r* and *Vpac2r*). Elucidation of the *in vivo* consequences of these diverse RAMP2-GPCR interactions is possible due to the availability of genetically engineered mouse models [1]. For example, global deletion of *Ramp2* in mice causes embryonic lethality by e14.5 due to proliferative defects in lymphatic vascular development [10, 11]. Because these *Ramp2* null phenotypes precisely mirror those observed in *Adm* and *Calcrl* knockout embryos, we and others have used this phenotypic conservation as *in vivo* validation substantiating the essential role of the canonical RAMP2-CLR interaction in vascular development. Interestingly, subsequent endothelial restoration of *Ramp2* expression is sufficient to partially rescue the *Ramp2*^*-/-*^ lethality but not without consequence, since surviving animals develop dilated cardiomyopathy associated with profound dysregulation *Gcgr* and *Pth1r* expression [12], indicating that genetic deletion of *Ramp2* can impart physiological consequences that extend beyond canonical AM/CLR signaling. Furthermore, adult female mice heterozygous for *Ramp2* demonstrate a constellation of endocrine abnormalities, including fetal growth restriction and hyperprolactinemia that are not phenotypically conserved in *Adm*^*+/-*^ and *Calcrl*^*+/-*^ female animals [13].

Here, we seek to expand our overall understanding of the numerous GPCR pathways impacted by RAMP2 by capitalizing on the mid-gestation *Ramp2*^*-/-*^ placenta—which fortuitously provides a phenotypically-tractable endocrine tissue with homozygous loss of RAMP2 expression [10]. The maternal-fetal interface of the placenta, called the labyrinth layer in rodents, consists of a tightly interdigitated network of trophoblast-lined fetal and maternal blood sinuses that support gas, nutrient, and waste exchange. Maternal blood is delivered to the labyrinth via high capacitance, low resistance maternal vessels called spiral arteries, which undergo an immune cell-mediated remodeling during mid-gestation in order to meet the demands of the growing embryo. Failure of spiral arteries to remodel at mid-gestation is associated with preeclampsia, a common hypertensive disease of pregnancy, which can lead to both fetal growth restriction and intrauterine fetal demise [14]. We hypothesize that, given the canonical RAMP2-CLR/AM signaling paradigm, deletion of *Ramp2* in the placenta will phenocopy *Calcrl*^*-/-*^ and *Adm*^*-/-*^ placentas and may further reveal additional phenotypes consistent with deletion of other RAMP2-associated GPCRs. Thus, a comprehensive characterization of the phenotypic effects of *Ramp2* loss within the placenta may elucidate additional RAMP2-associated GPCRs that play critical roles in systemic endocrine physiology and disease.

## Materials and methods

### Animals

Mice with a deletion of *Ramp2* were previously generated by our group and maintained as a heterozygote colony on an isogenic SvEv129/6-TC1 background [10]. Genotyping of the wild type and targeted *Ramp2* genes was also described previously [10]. All animal experiments were approved by the University of North Carolina at Chapel Hill Institutional Animal Care and Use Committee (Protocol No. 15–209). *Ramp*^*+/-*^ intercrosses were set up to generate *Ramp2*^*+/+*^ and *Ramp2*^*-/-*^ littermate placentas, which were used in all studies. Embryonic and therefore placental viability were confirmed visually at the time of dissection prior to collection of placentas for phenotypic analysis. Visualization of the vaginal plug was considered embryonic day (e) 0.5. Prior to dissection, animals were euthanized by carbon dioxide inhalation followed by cervical dislocation.

### PTH challenge

Five to seven week-old wild type and *Ramp2*^*+/-*^ female mice were injected with a single bolus of 500 μgkg^-1^ hPTH, and blood was collected 30 minutes and two hours later. Serum phosphate levels were determined using Stanbio kits (Fisher Scientific).

### Immunofluorescence

All tissues were fixed overnight in 4% paraformaldehyde, cryoprotected overnight in 30% sucrose, embedded in optical cutting temperature compound (Tissue-Tek), and serially sectioned at 8–10 μm. Sections were rehydrated in PBS, permeablized in 0.2% Triton-X100, blocked in 4% bovine serum albumin, and incubated overnight in primary antibody: rabbit polyclonal anti-cytokeratin (Dako, Z0622, AB_2650434); mouse monoclonal anti-BrdU (Invitrogen, 03–3900, AB_2532917); rat monoclonal anti-Ki-67 (Dako, M7249, AB_2250503); mouse monoclonal anti α-smooth muscle actin (Sigma, A2547, AB_476701); or mouse polyclonal parathyroid hormone receptor 1 (Novus, NBP1-40067, AB_2173351). After washing, sections were incubated in secondary antibody for 1–2 hours. Images were acquired on a Nikon E800 microscope with a Hammamatsu ORCA-ER charge-coupled device camera with MetaMorph software (Molecular Devices).

### *In situ* hybridization

e13.5 placentas from wild type 129S6/SvEv intercrosses were collected and processed as described for immunofluorescence, with the exception that paraformaldehyde and sucrose solutions were prepared in diethylypyrocarbonate-treated PBS. Placentas were serially sectioned at 16 μm. Non-radioactive ISH was performed as previously described using sense and anti-sense RNA probes synthesized from plasmids containing cDNA sequences of mouse *Ramp2* [15].

### qRT-PCR

RNA was extracted using TRIzol (Invitrogen) per the manufacturer’s protocol. RNA was DNase treated and reverse transcribed using M-MLV Reverse Transcriptase (Invitrogen), and gene expression was measured using a MX3000 qPCR machine (Stratagene). TaqMan primer and probes sets (Applied Biosystems) were used to measure gene expression. *mGapdh* was used as an endogenous reference gene, and relative gene expression was calculated using the ΔΔC_T_ method.

### Western blotting

Placental lysates were isolated and then quantitated for protein concentration using a BCA Protein Assay Kit (Pierce). Protein was loaded on a 4–12% SDS-PAGE gel (Invitrogen) and then transferred to a nitrocellulose membrane. The membrane was blocked overnight at 4°C in 5% nonfat dry milk and then incubated overnight at 4° in primary antibodies: rabbit polyclonal anti-PTH1R (Covance, PRB-620B) and mouse monoclonal GAPDH (Novus, NB300-285, AB_2263090). Blots were washed three times in tris-buffered saline/0.1% Tween 20 and then incubated for two hours at room temperature in secondary antibodies. Membranes were then imaged on an Odyssey CLx (LI-COR).

### Statistics

All data are presented as mean ± standard error of mean unless otherwise noted. All quantitative analyses were performed in GraphPad Prism 5 using unpaired two-tailed Student’s t-test. A *p* value less than 0.05 was considered statistically significant.

## Results

### *Ramp2* is abundantly expressed in the labyrinth of the placenta

**Fig 1:**
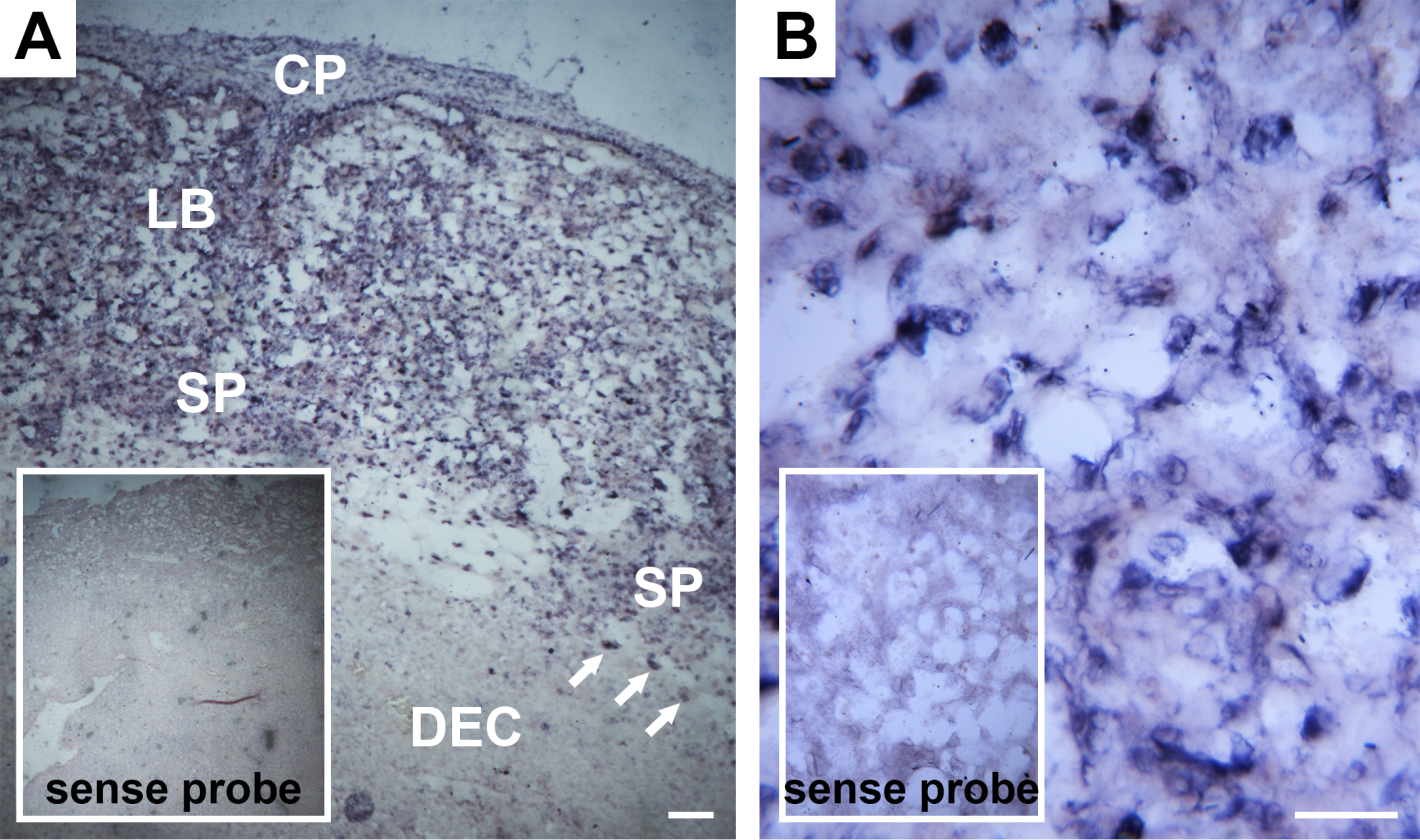
*In situ* hybridization of *Ramp2* in e13.5 placentas reveals robust expression in the labyrinth layer. (A) *In situ* hybridization in both the fetal and maternal compartments of the wild type placenta (n = 4). Scale bar, 100 μm. (B) *In situ* hybridization in the labyrinth layer. Scale bar, 50 μm. Inset images in both panels show sense control probes. CP, chorionic plate; LB, labyrinth; SP, spongiotrophoblast cells; DEC, decidua. Arrows point to giant trophoblast cells.

To characterize the expression and localization of *Ramp2* in the mouse placenta, we performed *in situ* hybridization for *Ramp2* in e13.5 wild type placentas. We observed expression of *Ramp2* in the fetal compartments of the placenta but only diffuse, weak signal in the maternal decidua (Fig 1A). More specifically, *Ramp2* expression was evident in the chorionic plate, labyrinth, spongiotrophoblast cells, and giant trophoblast cells, with the labyrinth layer demonstrating the most robust expression (Fig 1B).

### *Ramp2*^*-/-*^ placentas exhibit abnormal development of the labyrinth layer

**Fig 2:**
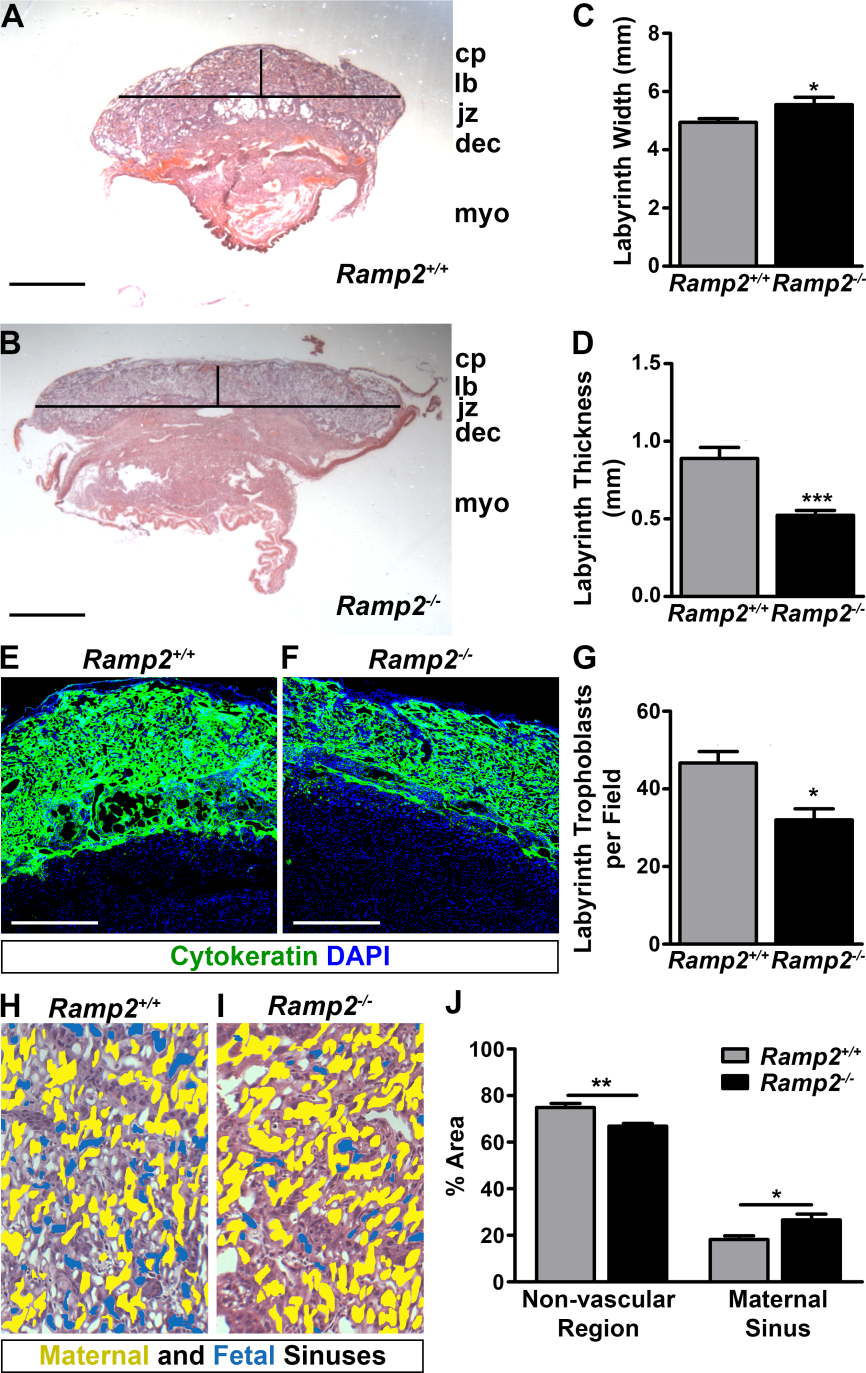
*Ramp2* is essential for proper development of the labyrinth layer of the placenta. (A and B), Hematoxylin and eosin staining of e13.5 wild type and *Ramp2*^*-/-*^ littermate placentas. Horizontal and vertical lines are representative of axes measured in panels (C) and (D) Scale bars, 1 mm. (C and D), Quantification of width and thickness of wild type and *Ramp2-/-* labyrinth layers (n≥6 placentas per genotype). (E and F), Immunofluorescence for cytokeratin in wild type and *Ramp2*^*-/-*^ placentas, showing trophoblast cells in the fetal compartment of the placenta. Scale bar, 500**μ**m. (G) Quantitation of labyrinth trophoblast cells, identified by histology (n = 3 fields per animal, n≥3 placentas per genotype). (H and I) Representative images of wild type and *Ramp2*^*-/-*^ placentas with pseudocolored maternal (yellow) and fetal (blue) sinuses. (J) Comparison of the total area of the non-vascular region and of the maternal sinuses in wild type and *Ramp2*^*-/-*^ placentas (n≥2 fields per placenta from multiple placentas per genotype). cp, chorionic plate; lb, labyrinth; jz, junctional zone; dec, dedicua; myo, myometrium. *p<0.05, **p<0.01, ***p<0.001

To evaluate the consequence of genetic loss of *Ramp2* on placental development, we performed histological analyses of e13.5 wild type and *Ramp2*^*-/-*^ littermate placentas obtained from *Ramp2*^*+/-*^ intercrosses. All layers of the placentas of both genotypes were visible by hematoxylin and eosin staining (Fig 2A and 2B). However, quantitative, morphometric analysis of these sections revealed a significantly wider, thinner labyrinth layer in *Ramp2*^*-/-*^ placentas (Fig 2C and 2D). We hypothesized that this thin labyrinth layer could be a consequence of fewer trophoblast cells. Therefore, we performed immunofluorescence on e13.5 placentas for the trophoblast marker cytokeratin, finding a reduction in the density of trophoblast cells in *Ramp2*^*-/-*^ placentas compared to wild type controls (Fig 2E and 2F). We also quantified labyrinth trophoblast cell density from e13.5 wild type and *Ramp2*^*-/-*^ placentas by histological identification. Indeed, *Ramp2*^*-/-*^ placentas had significantly fewer trophoblasts in the labyrinth layer compared to wild type littermates (Fig 2G).

We then evaluated the morphological consequences of labyrinth formation in *Ramp2*^*-/-*^ placentas, distinguishing between maternal and fetal sinuses by the presence or absence of nucleated red blood cells, respectively. Consistent with decreased trophoblast cellularity in the labyrinth of *Ramp2*^*-/-*^ placentas, the total area of non-vascular regions identified by this method was significantly less in *Ramp2*^*-/-*^ placentas compared to wild type placentas (Fig 2H–2J). Concomitantly, the interdigitated footprint of maternal sinuses was significantly greater in *Ramp2*^*-/-*^ placentas, indicating an improper balance between the fetal and maternal sinuses within the labyrinth layer. Altogether, these data point to a role for *Ramp2* in the proper formation of the maternal-fetal interface within the labyrinth layer.

### *Ramp2* is required for proliferation of trophoblast cells

**Fig 3:**
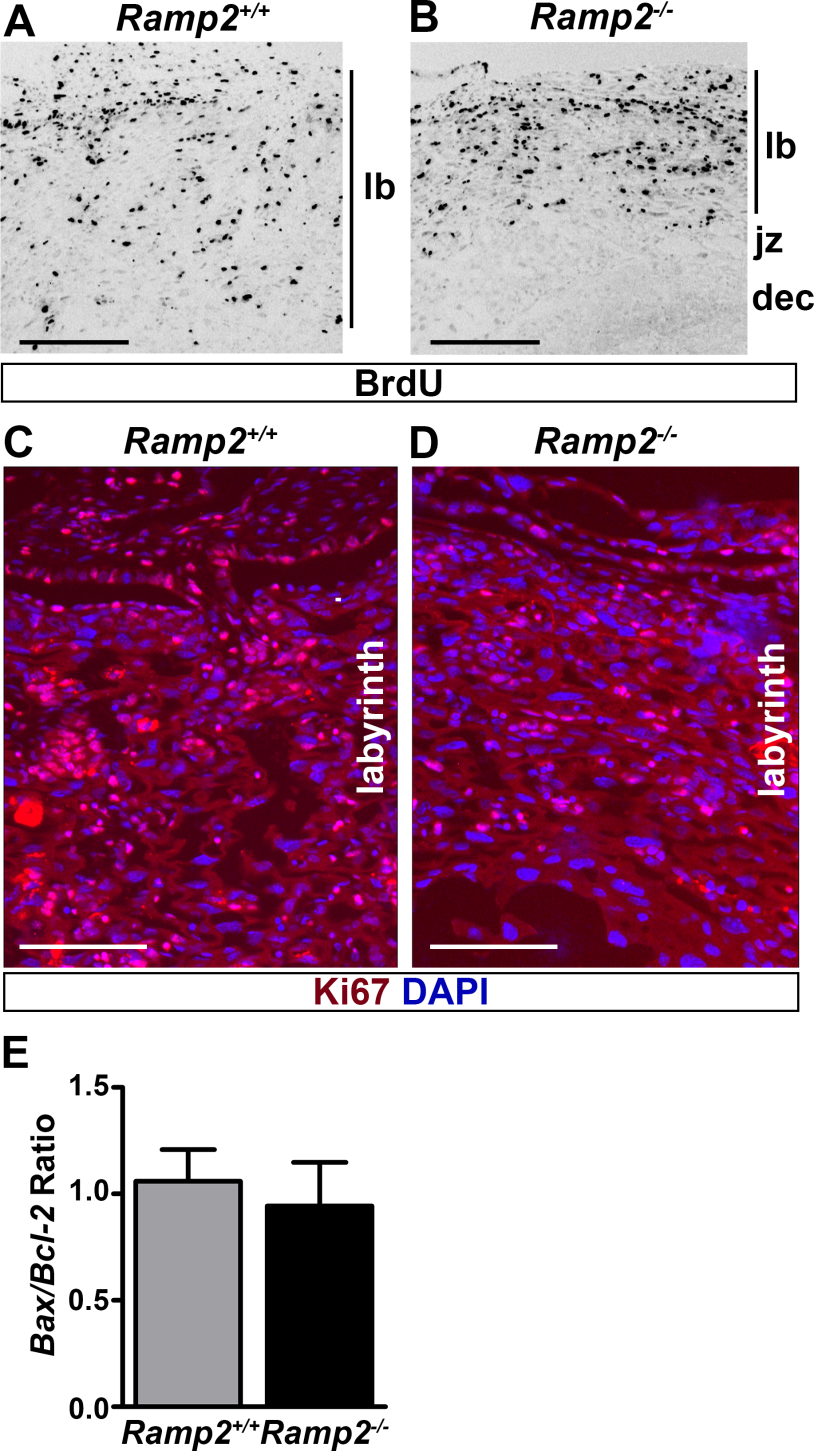
Loss of *Ramp2* leads to a proliferation defect in the labyrinth layer. (A and B) Immunofluorescence for BrdU in e13.5 placentas. Scale bars, 500**μ**m. (C and D) Immunofluorescence for Ki67 in the labyrinth of e13.5 placentas. Scale bars, 500 μm. (E) Ratio of *Bax* to *Bcl2* mRNA expression normalized to *Gapdh* as measured by qRT-PCR (n≥5 placentas per genotype). lb, labyrinth; jz, junctional zone; dec, decidua.

To determine if the decreased labyrinth thickness and trophoblast cell number were due to deficient trophoblast cell proliferation, we performed bromodeoxyuridine (BrdU) incorporation studies in pregnant *Ramp2*^*+/-*^ mice bred with *Ramp2*^*+/-*^ males on day 13.5 of gestation. BrdU staining revealed a marked reduction in cell proliferation in the labyrinth layer of *Ramp2*^*-/-*^ placentas compared to controls (Fig 3A and 3B). Consistently, Ki67 staining also demonstrated reduced signal in the labyrinth of *Ramp2*^*-/-*^ placentas compared to wild type littermate placentas, underscoring the important role of *Ramp2* in proliferation of trophoblast cells of the labyrinth layer (Fig 3C and 3D). To determine whether an enhanced apoptotic rate contributes to the observed decrease in trophoblast cellularity, we performed qRT-PCR to quantitate the expression of pro-apoptotic *Bax* and anti-apoptotic *Bcl2*. The ratio of *Bax* to *Bcl2* did not differ between the two genotypes, indicating that loss of *Ramp2* does not promote apoptosis of trophoblast cells (Fig 3E).

### Loss of *Ramp2* results in failed spiral artery remodeling

**Fig 4:**
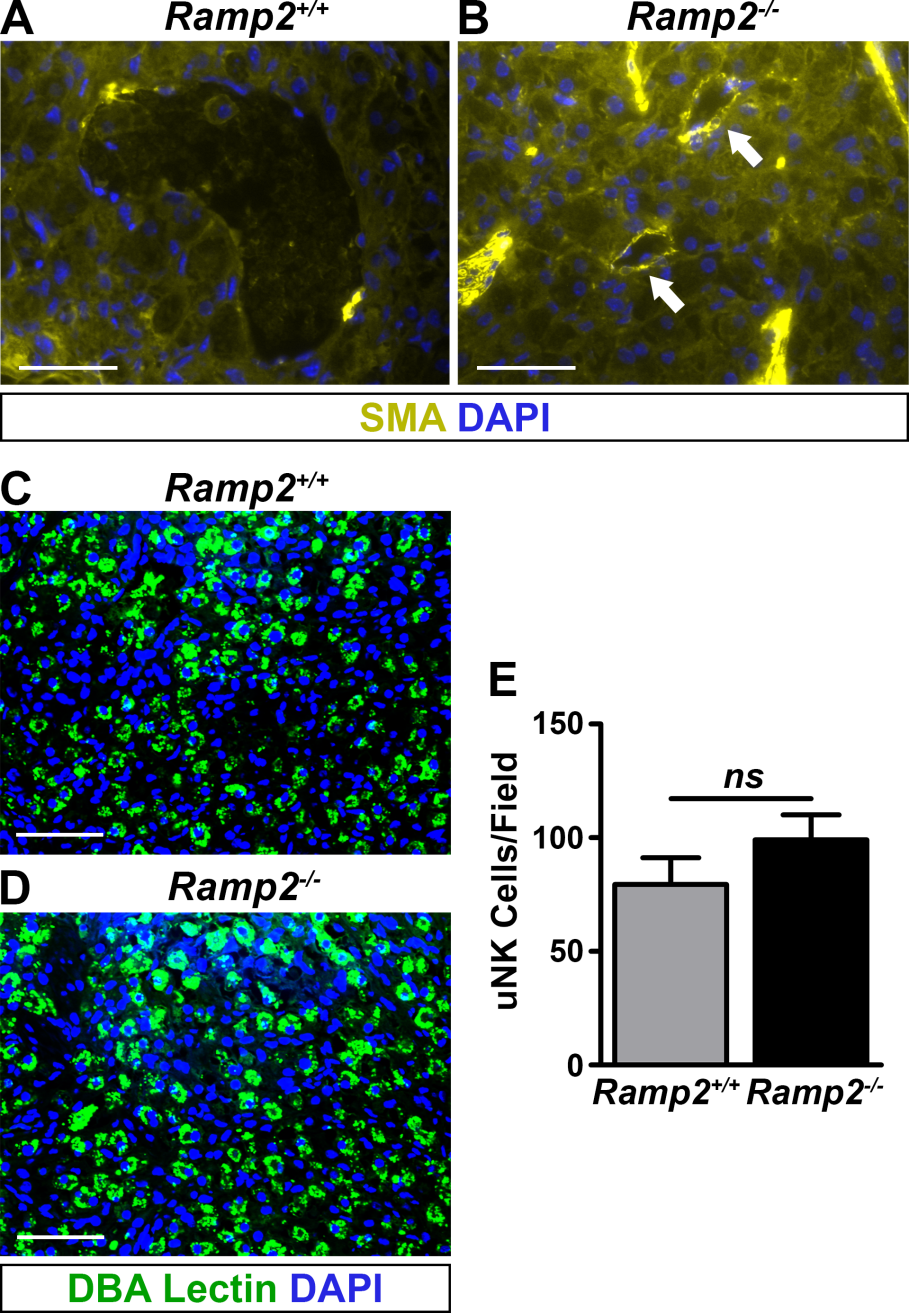
*Ramp2* is essential for proper spiral artery remodeling. (A and B), Immunofluorescence for smooth muscle actin in e13.5 littermate placentas (n≥6 placentas per genotype). Arrows point to retained smooth muscle actin expression in spiral arteries of *Ramp2*^*-/-*^ placentas. Scale bars, 100 μm. (C and D), Immunofluorescence for DBA lectin, a marker of uNK cells, in e13.5 littermate placentas. Scale bars, 100**μ**m. (E) Quantification of uNK cells from e13.5 placentas (n≥3 placentas per genotype). ns, not significant.

Maternal spiral artery remodeling, which consists of immune cell-mediated vascular smooth muscle cell loss and vessel dilation, is critical for maintaining appropriate placental function and promoting fetal health in both rodents and humans [16]. Therefore, we evaluated the extent of vascular smooth muscle coverage surrounding the maternal spiral arteries by performing immunofluorescence for smooth muscle actin on wild type and *Ramp2*^*-/-*^ placentas. As expected, spiral arteries of wild type placentas progressively lost smooth muscle coverage until mid-gestation to accommodate the vascular demands of the growing embryo (Fig 4A). However, we observed persistent smooth muscle coverage of the spiral arteries of *Ramp2*^*-/-*^ placentas (Fig 4B).

A specialized immune cell population in the placenta, uterine natural killer (uNK) cells, release factors that promote spiral artery remodeling [17–19]. We investigated the status of the uNK cell population in these placentas by performing immunofluorescence on e13.5 wild type and *Ramp2*^*-/-*^ littermate placentas and found no difference in the size of the uNK cell population, suggesting that the failure to remodel maternal spiral arteries in *Ramp2*^*-/-*^ placentas is inherent to the fetal genotype (Fig 4C–4E).

### Effect of *Ramp2* loss on gene expression of RAMP2-interacting GPCRs

**Fig 5:**
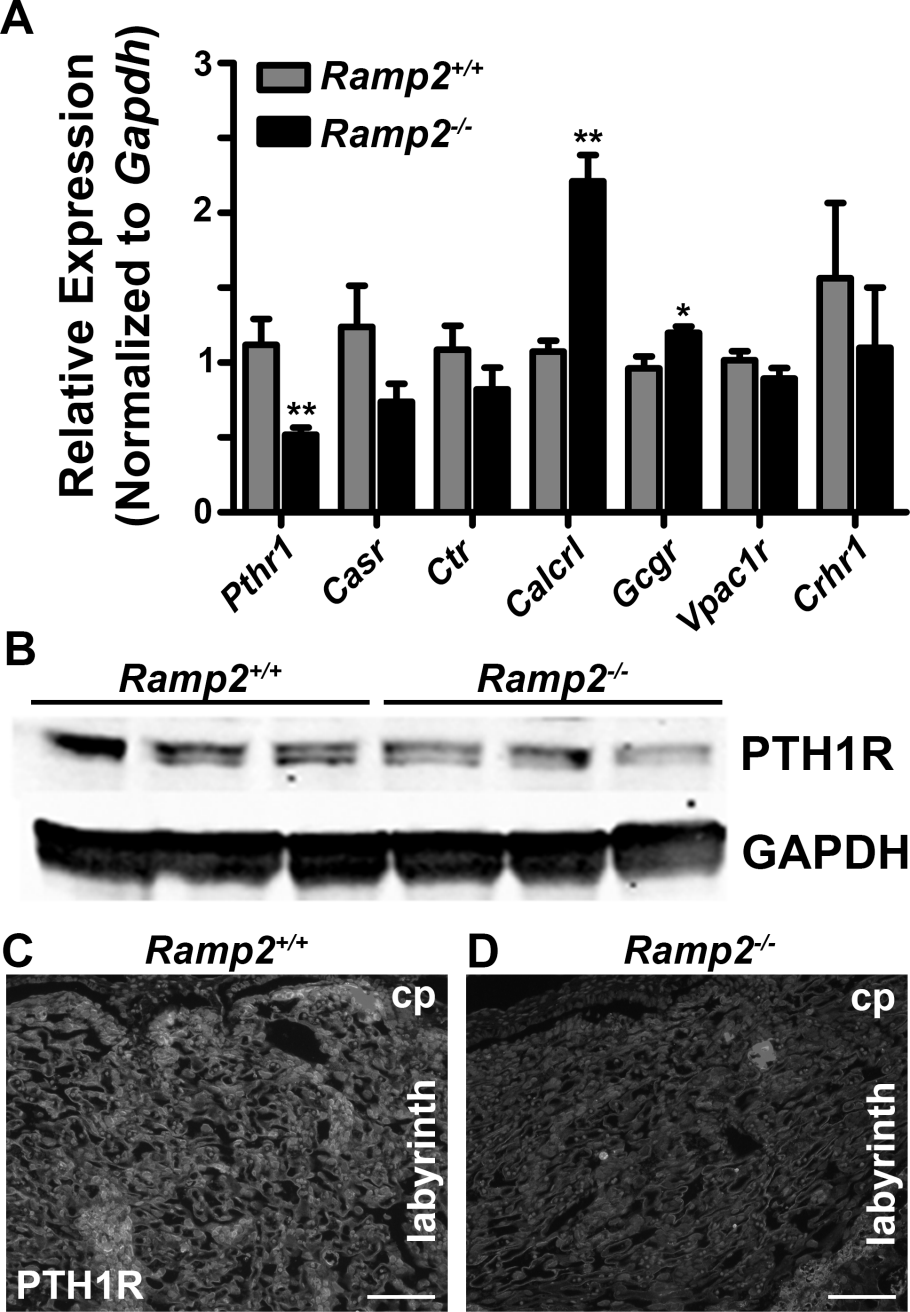
Loss of *Ramp2* alters mRNA expression of RAMP2-associating GPCRs, including *Pthr1*. (A) mRNA expression of RAMP2-interacting GPCRs from e13.5 wild type and *Ramp2*^*-/-*^ placentas (n≥3 placentas per genotype). (B) PTH1R protein expression by immunoblot of e13.5 tissue lysates from wild type and *Ramp2*^*-/-*^ placentas. (C and D), Immunofluorescence for PTH1R in the labyrinth of wild type and *Ramp2*^*-/-*^ placentas. Scale bars, 100 μm. *Pth1r*, parathyroid hormone receptor 1; *Casr*, calcium-sensing receptor; *Ctr*, calcitonin receptor; *Calcrl*, calcitonin receptor-like receptor; *Gcgr*, glucagon receptor; *Vpac1r*, vasoactive intestinal peptide receptor 1; *Crhr1*, corticotrophin releasing hormone receptor 1. *p<0.01.

We next attempted to elucidate candidate GPCRs that may be involved in the *Ramp2*^*-/-*^ placenta phenotype by performing qRT-PCR for a panel of known RAMP2-interacting GPCRs. We found a robust and significant increase in *Calcrl* gene expression, consistent with the retention of *Calcrl*-enriched smooth muscle cells surrounding the spiral arteries [20]. We also noted a modest yet significant increase in glucagon receptor. Most significantly, we found a 50% reduction in the expression of *Pthr1*, the receptor for parathyroid hormone (PTH) and parathyroid hormone-related protein (PTHrP). To determine whether the reduction of *Pth1r* mRNA correlated with reduced protein expression, we determined the levels of PTH1R in *Ramp2*^*-/-*^ knockout placentas using both western blotting and immunofluorescence. Consistently, we observed a reduction in protein levels in placental lysates by immunoblotting (Fig 5B). Furthermore, immunofluorescence for PTH1R confirmed that this receptor is highly expressed in the labyrinth layer and that *Ramp2*^*-/-*^ placentas had considerably less staining compared to wild type placentas (Fig 5C and 5D). Our results provide compelling evidence that loss of *Ramp2* alters *Pth1r* gene and protein expression in the placenta and specifically within the labyrinth layer.

### Haploinsufficiency for Ramp2 blunts PTH1R response to PTH

**Fig 6:**
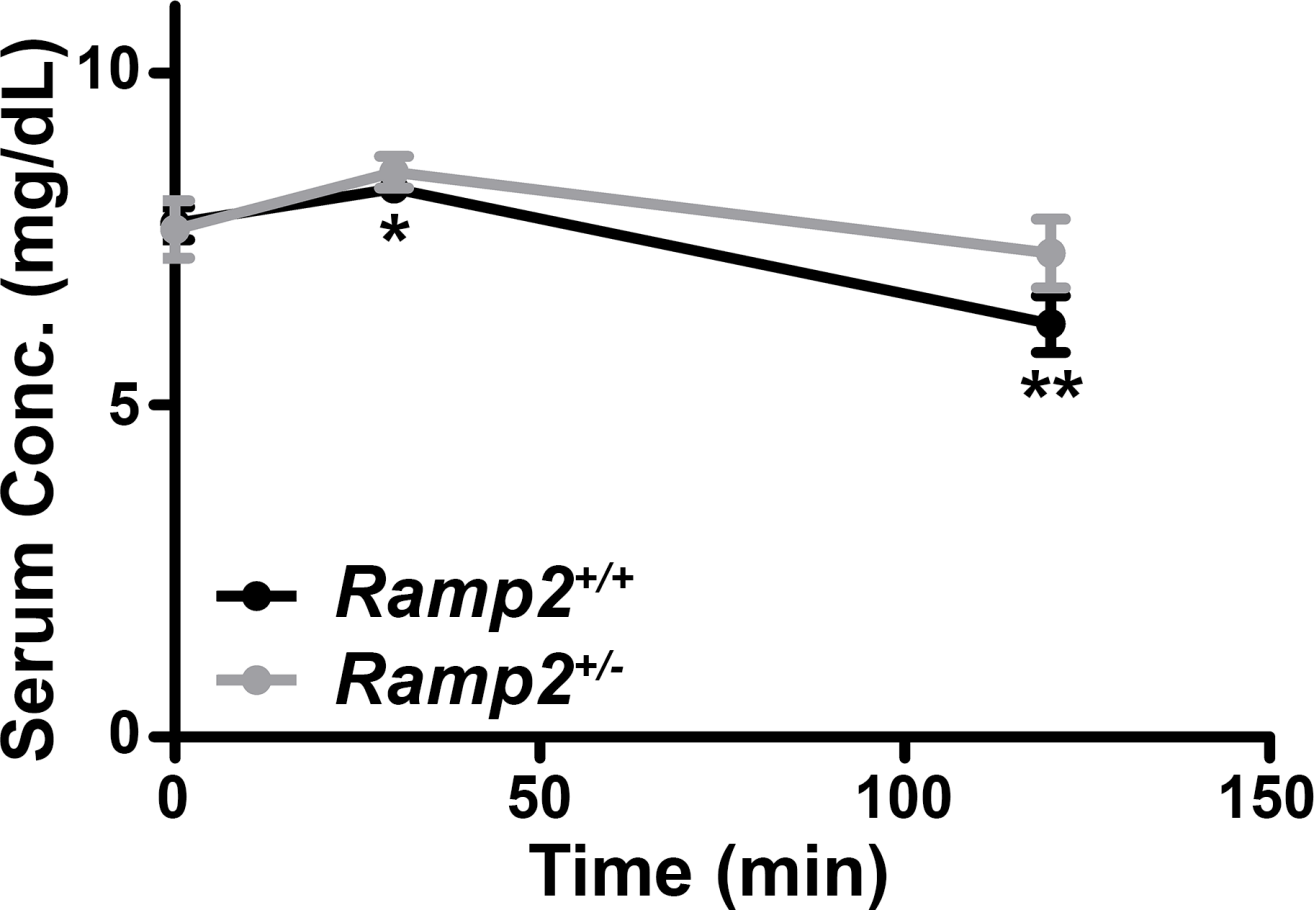
*Ramp2*^*+/-*^ adult mice demonstrate a blunted physiological response to systemic PTH administration. Serum concentrations of phosphate (solid lines) in wild type and *Ramp2*^*+/-*^ animals following administration of a single bolus of PTH (n≥5 animals per genotype). *p<0.05, **p<0.01.

Given changes in *Pthr1* expression in the context of *Ramp2* loss, we hypothesized that adult mice haploinsufficient for *Ramp2* may demonstrate a blunted physiological response to exogenous PTH administration. Indeed, exogenous PTH led to a significant reduction in serum phosphate levels in wild type mice, but this reduction did not occur in *Ramp2*^*+/-*^ animals (Fig 6). Therefore, these data indicate that haploinsufficiency for *Ramp2* results in blunted, systemic PTH1R signaling.

## Discussion

In this study, we have localized *Ramp2* within the mouse placenta, assessed the effect of *Ramp2* loss on placental development and discovered associated changes in the expression of RAMP2-interacting GPCRs with physiological consequences *in vivo*. First, we found *Ramp2* expressed primarily in the labyrinth of the e13.5 placenta. Interestingly, this expression pattern differs from the expression patterns of *Calcrl* and *Adm* at a similar developmental time points: at e13.5, *Calcrl* is diffusely expressed in the labyrinth but robustly expressed in endothelial cells lining spiral arteries, while *Adm* is primarily found in trophoblast giant cells and in the stroma of the decidua [20]. Furthermore, although *Adm*^*-/-*^ and *Calcrl*^*-/-*^ placentas consistently demonstrate decreased fetal vessel branching and other placental abnormalities, the structure of the labyrinth layer in these mouse models remains largely normal [20]. Therefore, these juxtaposed findings support the overall hypothesis that RAMP2 influences labyrinth development through association with another known or unknown GPCR.

We also found that *Ramp2*^*-/-*^ placentas–similar to *Adm*^*-/-*^ and *Calcrl*^*-/-*^ placentas–exhibit persistent smooth muscle cell coverage of maternal spiral arteries–a hallmark sign of preeclampsia, a dangerous hypertensive disease of pregnancy [21]. Consistently, one human study found that *Ramp2* mRNA expression in the umbilical artery and uterus of women was negatively correlated with pregnancy-induced hypertension [22]. Unlike *Adm*^*-/-*^ and *Calcrl*^*-/-*^ placentas, the size of the uNK cell population within the decidua was unchanged in *Ramp2*^*-/-*^ placentas. Interactions between uNK cells and trophoblast cells help facilitate spiral artery remodeling during mid-gestation as spiral arteries become high capacitance, low resistance vessels to provide oxygen and nutrients to the developing fetus [14]. Although pregnant *Ramp2*^*+/-*^ dams carrying *Ramp2*^*-/-*^ embryos do not develop overt preeclampsia, likely due to embryonic lethality of *Ramp2*^*-/-*^ embryos, our studies demonstrate that RAMP2 plays a role in spiral artery remodeling distinct from uNK cell recruitment and canonical AM signaling through RAMP2-CLR.

Given evidence for RAMP2-mediated effects on placental development independent of CLR, we analyzed changes in gene expression of all known RAMP2-associated GPCRs in *Ramp2*^*-/-*^ placental lysates, finding that *Pthr1* mRNA expression was approximately 50% lower by qRT-PCR compared to wild type littermate placentas. Elegant studies performed by Kovacs and colleagues previously identified roles for PTH1R ligands PTH and PTHrP in placental calcium transfer and trophoblast cell proliferation [23–26]. Consistent with these studies, we found that PTH1R expression was decreased in *Ramp2*^*-/-*^ placentas by immunoblotting and by immunofluorescence. We further hypothesized that *Ramp2*^*+/-*^ adult females would demonstrate a blunted response to systemic PTH administration. Indeed, systemic PTH caused a decrease in serum phosphate levels only in wild type females, not in *Ramp2* heterozygous females. While the long-term physiologic consequences of this are unclear, the established therapeutic roles of PTH and PTHrP in diseases like osteoporosis and cancer underscore the importance of understanding consequences of the RAMP2-PTH1R interaction in various organ systems *in vivo* [27–30].

In summary, the present study capitalizes on the availability of genetically deficient *Ramp2*^*-/-*^ tissue to provide insight into RAMP2-mediated effects on placental development and its possible role in the pathogenesis of preeclampsia, likely both in concert with and independent from canonical AM-CLR signaling. We further show that genetic loss of *Ramp2* causes a significant reduction in PTH1R, with physiological consequences in both the placenta and broader systemic endocrine system. Although our current study focused on the cadre of currently identified RAMP2-associating GPCRs, it is likely that there are additional GPCRs to be identified, providing additional lenses through which *in vivo* data on RAMP2 genetic models could be interpreted.
